# Targeting lung cancer screening to individuals at greatest risk: the role of genetic factors

**DOI:** 10.1136/jmedgenet-2020-107399

**Published:** 2021-01-29

**Authors:** Mikey B Lebrett, Emma J Crosbie, Miriam J Smith, Emma R Woodward, D Gareth Evans, Philip A J Crosbie

**Affiliations:** 1 Division of Infection, Immunity and Respiratory Medicine, The University of Manchester Faculty of Biology Medicine and Health, Manchester, UK; 2 Prevention and Early Detection Theme, NIHR Manchester Biomedical Research Centre, Manchester, UK; 3 Division of Cancer Sciences, The University of Manchester Faculty of Biology Medicine and Health, Manchester, UK; 4 Manchester Centre for Genomic Medicine, St Mary’s Hospital, Division of Evolution and Genomic Sciences, School of Biological Sciences, University of Manchester, Manchester, UK; 5 Manchester Thoracic Oncology Centre, Wythenshawe Hospital, Manchester University NHS Foundation Trust, Manchester, UK

**Keywords:** polymorphism, genetic, early diagnosis, genetic predisposition to disease, germ-line mutation

## Abstract

Lung cancer (LC) is the most common global cancer. An individual’s risk of developing LC is mediated by an array of factors, including family history of the disease. Considerable research into genetic risk factors for LC has taken place in recent years, with both low-penetrance and high-penetrance variants implicated in increasing or decreasing a person’s risk of the disease. LC is the leading cause of cancer death worldwide; poor survival is driven by late onset of non-specific symptoms, resulting in late-stage diagnoses. Evidence for the efficacy of screening in detecting cancer earlier, thereby reducing lung-cancer specific mortality, is now well established. To ensure the cost-effectiveness of a screening programme and to limit the potential harms to participants, a risk threshold for screening eligibility is required. Risk prediction models (RPMs), which provide an individual’s personal risk of LC over a particular period based on a large number of risk factors, may improve the selection of high-risk individuals for LC screening when compared with generalised eligibility criteria that only consider smoking history and age. No currently used RPM integrates genetic risk factors into its calculation of risk. This review provides an overview of the evidence for LC screening, screening related harms and the use of RPMs in screening cohort selection. It gives a synopsis of the known genetic risk factors for lung cancer and discusses the evidence for including them in RPMs, focusing in particular on the use of polygenic risk scores to increase the accuracy of targeted lung cancer screening.

## Introduction

Lung cancer is the leading cause of cancer death worldwide. It is the most common cancer in men and the third most common cancer in women.^1^ There were an estimated 2.1 million new cases and 1.8 million deaths in 2018, representing almost 12% of all cancer diagnoses and 18% of cancer deaths globally. In the UK, 47 000 new cases are diagnosed each year,^2^ and it is responsible for one in five cancer deaths.^3^ Lung cancer is classified into two main types, non-small cell lung cancer (NSCLC) and small cell lung cancer (SCLC). NSCLC is responsible for approximately 85% of cases and is composed of a number of histological subtypes, most commonly adenocarcinoma, squamous cell carcinoma and large cell carcinoma.^4^ While less common, SCLC is more aggressive than NSCLC, with faster doubling times and a higher tendency to metastasise at an earlier stage.^5^

Smoking and age are the two most important risk factors for lung cancer. In the UK, smoking is estimated to cause up to 86% of cases.^6 7^ This risk rises with both smoking duration and number of cigarettes smoked each day.^8^ Just under half of cases occur in people aged over 75 years. The highest rate of lung cancer occurs between the ages of 80 and 84 years in women and 85–89 years in men.^9^ Several other risk factors increase a person’s chance of developing lung cancer, particularly radon exposure,^10^ workplace exposure to asbestos and other harmful agents (responsible for ≈13% of UK case^6^), socioeconomic deprivation,^11^ previous diagnosis of a malignant tumour,^12^ previous diagnosis of respiratory conditions such as chronic obstructive pulmonary disease (COPD)^13–15^ (the evidence for pneumonia and tuberculosis is less well established^14 16 17^), family history,^18^ as well as particular rare hereditary disorders such as Li Fraumeni syndrome^19^ and the recently described non-syndromic association with germline EGFR mutation.^20^ The impact of female sex on lung cancer risk is debated.^8 21–23^

Lung cancer survival is poor. Overall, only 40% of patients in England and Wales survive for 1 year following diagnosis, with this proportion dropping to approximately 16% survival over 5 years and 10% over 10 years.^24^ While 1-year survival in England and Wales has increased significantly since the 1970s, long-term survival rates have only seen a modest improvement; for example, the 5-year age-standardised survival rate increased by just 4% for men and 7% for women between 1971 and 2011.^25^ This stands in stark contrast to the doubling of overall cancer survival in the UK over the past 40 years.^26^

The late clinical presentation of lung cancer is a major reason for its low survival rates and poor prognosis. In the UK, around half of patients have distant metastases and therefore incurable stage IV disease at the time of diagnosis compared with just a quarter with stage I or stage II disease.^27^ The 1-year survival rate of stage IV disease is 17%, compared with 83% for those diagnosed at stage I.^28^ Even within stage I, survival is predicted by tumour diameter. Five-year survival decreases by 5% for each 1 cm that tumour diameter increases; this emphasises the importance of early detection, even at the earliest stage of lung cancer development.^29^

The primary reason lung cancer is diagnosed at a late stage is that early stage disease is often asymptomatic, and when symptoms do develop, they are usually mild and non-specific resulting in diagnostic delay. For example, fatigue, shortness of breath, cough, chest pain and persistent chest infections are all common symptoms of lung cancer but are also symptoms of other smoking-related conditions, such as COPD, which commonly coexist in patients with lung cancer.^30^

The key to improving patient outcomes is early detection. Low dose CT (LDCT) screening for those at high risk detects early stage lung cancer and reduces lung cancer specific mortality. Risk prediction models (RPMs) are used to select a high-risk cohort for screening. Currently, RPMs do not include a direct measure of genetic risk as a variable, as the evidence for the significance of genetic risk factors in lung cancer is still emerging. Here, we summarise the evidence for screening and various methods of screening cohort selection, focusing in particular on the contribution of genetics to lung cancer risk, and thereby in the potential of using genetic factors in selecting individuals for screening.

## Evidence for the effectiveness of lung cancer screening

Several approaches to screening have been trialled over the years. A meta-analysis published by Cochrane in 2004 concluded that there is no benefit derived from chest X-ray (CXR) or sputum cytology for lung cancer screening, modalities that had predominated screening trials since the 1960s.^31^ This was confirmed in 2011 by the large Prostate, Lung, Colorectal, and Ovarian (PLCO) Cancer Screening Trial that randomised 154 901 participants into CXR and standard care arms; there was no reduction in lung cancer mortality in the CXR cohort.^32^ LDCT emerged as a superior alternative to CXR for lung cancer screening in the 1990s. In a Japanese study of 1369 high-risk individuals, LDCT successfully identified 15 cases of lung cancer, 11 of which were missed by CXR. Most of the screen-detected cancers were stage I.^33^ The International Early Lung Cancer Action Program was the first international multicentre LDCT programme, running from 1993 to 2005. The programme screened 31 567 high-risk individuals, 27 456 of which had a repeat screening 1 year after baseline. LDCT screening identified 484 lung cancers, 85% of which were stage I.^34^

Evidence of a disease-specific mortality reduction from lung cancer screening was first demonstrated by the USA-based National Lung Screening Trial (NLST). This large study randomised 53 454 current or former smokers (≥30 packyears, smoked within 15 years), age 55–74 years at recruitment, to either annual LDCT or CXR over three screening rounds. LDCT screening detected lung cancer at an earlier stage (50% stage I) compared with the CXR arm (31% stage I). This resulted in a 20% reduction in lung cancer specific mortality and 6.7% reduction in all-cause mortality.^35^ The Dutch-Belgian Randomised Lung Cancer Screening Trial (NELSON) randomised 13 195 men and 2594 women age 50–75 years to either four rounds of LDCT screening over 5.5 years or no screening. All participants were current or former smokers who had smoked within 10 years and had a tobacco exposure of either ≥15 cigarettes per day for 25 years or ≥10 cigarettes per day for 30 years. NELSON confirmed NLST findings in a European population, reporting a 26% reduction in lung cancer deaths among men and a 33% reduction in lung cancer deaths among women with LDCT screening after 10 years of follow-up.^36^ The German Lung Cancer Screening Intervention trial showed an even larger discrepancy between the screening benefits derived by male and female screening cohorts, with women experiencing a statistically significant reduction in lung cancer mortality that was not replicated among men.^37^ The recently published Multicentric Italian Lung Detection (MILD) trial (age 50–75 years, current or former smokers within 10 years, ≥20 pack-years) reported a 39% reduction in lung cancer mortality over 10 years due to LDCT screening.^38^

## Screening-related harms

While there is significant evidence for the benefit of lung cancer screening, potential harms of screening must be considered. Overdiagnosis occurs when tumours are detected through screening that have no clinical consequence. This may result in the patient undergoing invasive treatment, exposing them to unnecessary harms to remove a tumour that would never have shortened their life or impaired its quality.^39^ Overdiagnosis has affected lung cancer screening trials in the past; 16 years of follow-up to a historic screening trial of CXR and sputum cytology found a likely overdiagnosis rate of 51%.^40^ More recent LDCT screening trials have lower, although still significant, overdiagnosis rates. The NELSON trial reported an upper overdiagnosis rate of between 8.9% and 19.7% depending on the length of follow-up considered.^36^ One study initially estimated the overdiagnosis rate in NLST to be up to 18.5%^41^; however, with extended follow-up, this estimate is now reported to be approximately 3%.^42^

False-positive results (in which scan findings mandate further investigation with no eventual cancer diagnosis) can result in a notable, although transient, spike in anxiety as well as invasive and unnecessary investigations.^43 44^ Some studies have shown long-term negative psychosocial impacts of a false-positive screening result.^45^ Adverse events related to invasive diagnostic procedures such as CT-guided biopsies are a significant potential harm of screening, particularly in individuals who are eventually found not to have lung cancer; one meta-analysis reported a 38.8% overall complication rate and 5.7% major complication rate for core biopsies of the lung.^46^ Radiation exposure from LDCT scans is also a potential harm (although the actual increased risk is minimal^47^).

While mitigating screening-related harms is a complex and multifaceted exercise, improved precision of lung cancer risk prediction increases the overall risk profile of the screening cohort, thereby improving the risk-to-benefit ratio. For example, risk stratification in breast cancer screening has been shown to decrease the overdiagnosis rate by 27%.^48^

## Use of risk prediction models to select screening participants for LDCT

NLST, NELSON and MILD selected screening participants solely based on age and smoking history. These generalised eligibility criteria serve as a rudimentary risk threshold for screening but do not predict an individual’s personal risk of lung cancer. This resulted in a screening cohort with a heterogeneous mix of risk profiles. Retrospective analysis of the NLST cohort stratified by lung cancer risk demonstrated marked variation in screening benefits and harms. Only 1% of lung cancer deaths prevented by LDCT screening were found in the lowest risk quintile despite similar exposure to screening harms.^49^ The number needed to screen to prevent one death was 161 in the highest risk group and 5276 in the lowest. It has been proposed that replacing generic eligibility criteria with a personalised lung cancer RPM could be a more effective way of selecting high-risk individuals for screening by LDCT. RPMs use several variables to estimate a specific individual’s risk of developing a disease over a period of time. Selection in this manner results in a screening cohort with a higher risk profile, thereby improving cost-effectiveness and efficacy of the programme, as well as reducing screening-related harms.^50 51^

More than 20 RPMs have been created for lung cancer.^52^ The Liverpool Lung Project (LLP) model is a validated RPM which, in addition to age and tobacco smoke exposure, includes asbestos exposure, sex, previous pneumonia diagnosis and previous cancer diagnoses as variables.^23^ This RPM was used prospectively in the UK Lung Cancer Screening Trial, in which 4055 participants were selected (based on an individualised 5-year lung cancer risk score of ≥5%) and randomised into LDCT screening or standard care (no screening) groups. In the screening group, 42 participants were diagnosed with lung cancer (2.1%), 85.7% of which were stage I or stage II at the time of diagnosis.^53^ As well as showing the efficacy of RPM use for screening selection, the study also demonstrated that lung cancer screening, based on individual risk, could be cost-effective within a UK-based healthcare setting.

PLCO_M2012_ is a logistic regression model developed using the disease incidence data of more than 80 000 smokers taking part in the PLCO cancer screening trial.^8^ In addition to tobacco smoke exposure and age, PLCO_M2012_ considers deprivation level (using educational attainment as a surrogate marker), COPD diagnosis, ethnicity, family history of lung cancer, personal history of cancer and BMI as risk factors. It was initially validated using the NLST cohort and has since been validated in several other trials.^54^ Studies have shown that PLCO_M2012_ increases the proportion of people selected for screening who have lung cancer when compared with generalised eligibility criteria.^8 55^ The model was employed successfully to determine screening eligibility in the Manchester Lung Health Check Pilot. A total of 1429 high-risk individuals were screened in the context of a commissioned service within the National Health Service (NHS). The risk threshold for LDCT screening was PLCO_M2012_ ≥1.51%^56^; at this threshold, PLCO_M2012_ has been shown to have improved sensitivity, specificity and positive predictive value for lung cancer detection compared with NLST generalised eligibility criteria.^55^ Across the trial, 4.4% of the cohort was diagnosed with lung cancer, more than double the rate found by NLST.^57^ Retrospective analysis found that within the group of patients with cancer diagnosed, PLCO_M2012_ and LLP_v2_ at a ≥2.5% risk threshold would have outperformed NLST generalised eligibility criteria that would have missed 18% of cancers.^58^ Based on the success of this pilot, NHS England have commissioned a further 10 screening pilots across the country.^59^

Generalised eligibility criteria select screening participants based on the two main risk factors of lung cancer, tobacco smoke exposure and age. Relying solely on these variables presents a number of limitations. USPSTF generalised eligibility criteria recommend lung cancer screening be offered to those aged 55–80 years who are current smokers or who have quit in the past 15 years. Despite the immediate and considerable benefits of smoking cessation,^60^ a recent meta-analysis confirmed that ever-smokers maintain an increased level of lung cancer risk well after 15 years since quitting^61^; other studies have demonstrated increased lung cancer incidence even 25 years after quitting.^62^ Consequently, these selection criteria may exclude previous smokers who remain at a heightened risk of lung cancer. Additionally, while age is an accurate predictor of lung cancer, older populations display considerable heterogeneity in their health trajectories.^63^ Consequently, biological age is a better indicator of health outcomes than chronological age. However, biomarkers and clinical measures of biological age are not well developed.^64^ The supplementary risk factors considered in all RPMs assist in targeting screening to the most at-risk individuals, bypassing the limitations of establishing screening eligibility by smoking history and age alone. Germline genetic biomarkers may be particularly valuable in this regard, as unlike other risk factors, they stay constant throughout a person’s life and are not impacted by smoking history.

## Genetic factors and lung cancer risk

### Epidemiology

Having a first-degree relative who has been diagnosed with lung cancer increases a person’s risk of also developing the disease.^65^ An individual with multiple family members diagnosed with lung cancer is at even greater risk; early onset lung cancer in affected family members also increases personal risk.^18^ While shared environmental and lifestyle factors within families are certainly responsible for part of the increased familial lung cancer risk, research over the past few years has established that there is an important hereditary genetic contribution as well. A pooled analysis of over 24 000 lung cancer cases found that after controlling for smoking and other confounding environmental factors, there was a 1.51-fold increase in risk of lung cancer in those who had a first-degree relative with the disease. Individuals with a sibling diagnosed with lung cancer were found to be at the highest increased risk, even after controlling for tobacco exposure.^66^ It should be noted that this pooled analysis only includes case–control studies that may be affected by sampling bias. Whether the magnitude of the effect reported would be reproduced at a population level in a prospective cohort is unclear. Despite this limitation, the implication of a significant familial element to increased lung cancer risk, with a potential genetic contribution, remains strongly supported by this and other studies.

A large Icelandic study found that spouses of patients with lung cancer have a 1.75-fold increased risk of lung cancer, indicating that shared environment is an important factor in the development of lung cancer. The same study demonstrated that first-degree relatives had a greater risk, up to a 3.5-fold increase. Although this observation may be due to the interplay between environmental and genetic factors, the exact nature of this interaction and the mechanism of genetic susceptibility are not elucidated.^67^ A multicentre study found that the risk of lung cancer increases with family history of the disease even among non-smoking women, providing further evidence that there is an important genetic contribution.^68^ Similarly, another study showed that non-smoking relatives of never-smoker lung cancer patients have a higher risk of contracting the disease when compared with controls, even though tobacco smoke did not contribute.^69^ Increased genetic risk of lung cancer may be particularly vital when it comes to early onset lung cancer,^70^ as well as multiple primary lung cancers.^71^ A study of 230 never-smokers with lung cancer found that 18% had family history of the disease, and a large proportion had specific genetic pathogenic variants that increase an individual’s susceptibility to developing lung cancer.^72^ A large prospective twin-based study estimated that the overall heritability of lung cancer is 18%.^73^ Heritability refers to the limit of genetic risk stratification on a population level and individuals may have a much higher level of genetically conferred lung cancer risk.^74^ These studies demonstrate the importance of genetic variables in defining lung cancer risk.

### Monogenic variants

Although there is strong evidence for a familial component of lung cancer, there is limited evidence that pathogenic variants in single genes confer high risk of lung cancer. A notable exception is the rare cancer predisposition syndrome, Li Fraumeni syndrome, arising from germline TP53 pathogenic variants.^75^ While typically associated with sarcoma, breast and brain tumours, leukaemia, lymphoma and adrenocortical carcinoma, instances of lung cancer in Li Fraumeni syndrome have also been described.^76 77^

Considerable research has taken place in the past decades to search for other significant single-gene variants associated with lung cancer risk. Several rare inherited *EGFR* variants are associated with lung cancer risk^78^; while the mechanism through which these variants increase disease risk is not confirmed, one possibility might be that the mutation causes genetic instability that predisposes cells to somatic mutations and tumourigenesis^20^; for example, the T790M variant in *EGFR* is both a germline variant associated with lung cancer and an important somatic variant with implications for therapy.^79 80^ The rarity and unclear penetrance of germline *EGFR* mutations makes discovery and subsequent management challenging.

Segregation analyses of families with high lung cancer incidence has provided some evidence for the existence of a rare major autosomal inherited allele that could contribute to a significant increase in lung cancer risk in its carriers.^81–83^ This hypothesis was further supported by a linkage analysis study of 52 high-risk families indicating a locus containing an inherited high-penetrance allele significantly associated with lung cancer risk to chr6q.^84^ A further study published in 2010 supported the implication of this chromosomal region in increasing lung cancer risk, even in never smokers^85^; fine mapping implicated the gene *RGS17* within this region as a likely candidate for familial lung cancer susceptibility.^86^ While *RGS17* overexpression has been shown to aid tumour cell proliferation, it has not been convincingly proven as a lung cancer susceptibility gene.^87^ A study published in 2015 demonstrated that a high-penetrance missense mutation in the *YAP1* oncogene significantly increases the risk of lung cancer.^88^ Another reported association was with the c.823C>T (p.Arg275Trp) missense variant in *PARK2*.^89^ However, given its low allele frequency in gnomAD (<0.002) and its lack of subsequent validation, it appears unlikely to be a high-risk allele.^90^ Overall, while useful for explaining the occurrence of some familial cancer, the rarity of high-penetrance variants within the population limits their usefulness in the context of routine, prescreening risk prediction for selecting a cohort from the general population. Consequently, the search for high frequency, low-penetrance alleles associated with lung cancer has become a more promising endeavour in recent years.

### Polygenic variants

Most of the accumulated evidence is of a polygenic inheritance pattern. There is now a large array of low-penetrance genetic variants identified, which either increase or decrease lung cancer risk by small amounts. These variants are usually discovered in genome wide association studies (GWAS). In a GWAS, hundreds of thousands or millions of SNPs are genotyped with the aim of finding variants that are present at a significantly higher frequency in the case group when compared with the control group. An OR can then be calculated, indicating the likelihood of a particular outcome (lung cancer) based on the presence of an exposure (a particular genetic variant). Genome wide significance for an allele is usually established with a p value of less than 5×10^–7^.

Since 2008, there have been over 45 genetic loci associated with lung cancer risk discovered by many thousands of GWAS, although the strength of evidence varies in each case.^91^ Meta-analyses seek to synthesise the large volume of (often conflicting) evidence generated by GWAS and case–control studies to generate a list of SNPs with robust association with lung cancer risk. For example, a large meta-analysis published in 2017 examined 246 genetic variants from 138 loci sourced from more than 1000 publications published until 2015. The mean number of cases in the studies analysed was 414 (range 13–4257), with the mean number of controls being 565 (range 12–55 823). The meta-analysis concluded that 22 variants in 21 genes showed significant association with lung cancer with strong cumulative epidemiological evidence (as graded by the Venice Criteria^92^). It also found a significant level of heterogeneity between the SNPs associated with various subgroups, including ethnicity, lung cancer histology and smoking status.^93^ A large number of meta-analyses such as this have been published in recent years.^94^

A review published in 2017 aimed to summarise and assess the evidence relating to lung cancer associated SNPs from more than 200 separate meta-analyses and GWAS, all published up to 2016 with at least 1000 cases.^94^ The majority of studies contained two or more ethnicities, although nine were limited to a specific ethnicity (six Asian and three Caucasian). The mean total sample size was 23 000; the median was 10 551 (the range was 1095–150 256). In total, the study yielded 137 SNPs associated with lung cancer risk, 80 of which were statistically significant. SNPs derived from meta-analyses were graded for strength of evidence using Venice Criteria and false positive report probability^95^; of the variants derived from the meta-analyses, 15 SNPs were graded as ‘strong’ for evidence of association and 19 SNPs were graded as ‘moderate’. This review did not weigh and synthesise the evidence for each SNP as a formal meta-analysis would have; when there was conflicting evidence from different studies, the evidence from the largest study was treated as authoritative. Nevertheless, this study serves as an important summary of the SNPs likely to exhibit robust association with lung cancer.

Since the publication of these studies, a further large case–control study was published in 2017. A total of 14 803 lung cancer cases and 12 262 controls were genotyped and aggregated with existing data resulting in an analysis of 29 266 cases and 56 450 controls.^96^ This study reported the discovery of 10 novel SNPs significantly associated with lung cancer, as well as the confirmation of 8 SNPs previously reported. The study claims to identify the SNPs responsible for 12.3% of the additional familial relative risk of lung cancer.

A considerable number of SNPs associated with increased lung cancer risk in European populations are localised to three particular gene clusters.

#### *CHRNA*

The *CHRNA* gene cluster is located in the 15q25 chromosomal region; variants within this region are strongly associated with lung cancer risk. For example, AA risk genotype at rs16969968 in *CHRNA5* is associated with both an increased risk and earlier diagnosis of lung cancer.^97^ Expression of the gene has been found to contribute to cancer cell signalling, proliferation, inhibition of apoptosis and angiogenesis.^98^ Additionally, studies have identified *CHRNA5* as having a role in nicotine addiction and dependency.^99^ Several studies have demonstrated that increased lung cancer risk is an independent association related to SNPs in this gene.^100–102^ SNP rs1051730 in the *CHRNA* gene is a variant with significantly robust association with lung cancer risk in European populations.^94^

#### *CLPTM1L*

The *CLPTM1L* gene is located in the 5p15 chromosomal region; two variants (rs401681 and rs402710) are particularly strongly associated with increased lung cancer risk.^103^ It has been proposed that the gene segment containing these polymorphisms may regulate telomerase reverse transcriptase expression, allowing cells to resist apoptosis and become malignant.^104^

#### *BAT3*

The *BAT3* gene is located in the 6p21 chromosomal region. The protein product of this gene cluster has been shown to be crucial in p53 acetylation during the repair or apoptosis of damaged, potentially malignant, cells. BAT3 may also be released in response to stress signals, engaging natural killer cells to target tumour cells.^105^

## Using genetic risk factors to select individuals for screening

The potential for inclusion of genetic risk factors in RPMs to improve risk prediction has been demonstrated in several disease areas, most notably breast cancer. High-penetrance genetic variants have been include in the Tyrer-Cuzick, BOADICEA and BRCAPRO models for breast cancer risk prediction, which use *BRCA1/2* mutation carrier status as a risk factor.^106^ In recent years, research has demonstrated the efficacy of employing a polygenic risk score (PRS) of low-penetrance SNPs in disease risk prediction. A PRS combines a selection of SNPs known to influence an individual’s risk of developing a disease; while each SNP may only have a minimal impact individually, when combined, they can alter risk significantly. A recent study demonstrated the utility of a PRS of 313 SNPs in breast cancer risk prediction.^107^ There is also evidence that PRS usage could reduce overdiagnosis in prostate cancer screening programmes,^108^ as well as facilitate the stratification of colorectal cancer screening by risk.^109^ Use of a PRS has also been proposed for the identification of individuals at increased risk of cardiovascular disease^110^ and Alzheimer’s disease.^111^ A study of more than 81 000 individuals published in August 2020 demonstrated that polygenic and monogenic risk factors interact with each other to modify risk in breast cancer, coronary artery disease and colon cancer.^112^ A selection of polygenic variants can influence the level of penetrance of the monogenic risk factor; consequently, a PRS can be used to predict the level of increased risk conferred by the monogenic risk variant carried by the individual.

While several lung cancer RPMs (including PLCO_M2012_) consider family history of lung cancer as a risk factor, no widely used model includes a direct biological measure of genetic risk. Despite there not being a known common high-penetrance gene for lung cancer that could be integrated into an RPM (such as *BRCA1/2* in breast cancer, which is not associated with lung cancer^113^), there is evidence that a PRS of low-penetrance SNPs could have utility in lung cancer risk prediction and screening selection. The Young RPM, which was published in 2009, showed that including a PRS comprising of 20 SNPs associated with lung cancer risk increased the predictive ability of the RPM when compared with standard risk factors alone.^114^ However, the RPM was not externally validated in an independent population and had certain non-standardised study design elements.^115^ Two further studies demonstrated that the incorporation of individual genetic markers into lung cancer RPMs (Improved LLP and Expanded Spitz) improved predictive ability by modest amounts.^116–118^ Crucially, the development of all three of these models preceded the large-scale meta-analyses published in recent years, which provide the best evidence for which SNPs are most robustly associated with lung cancer risk in large and diverse populations. Consequently, while serving as important proofs of concept, these RPMs are of limited clinical utility.

More recent case–control studies again demonstrated that the inclusion of selected SNPs in models can improve lung cancer risk prediction.^119–121^ While these improvements were often too small to have a major benefit in the context of a screening programme, this is primarily a result of the small SNP panels tested. Successful PRS systems rely on the combination of a very large number of independent SNPs from a range of loci^122^; with the increase in meta-analyses of GWAS seen in the past few years (thereby increasing the pool of potentially predictive SNPs), the prospect of a successful integration of lung cancer risk predictive SNPs into a RPM becomes ever more possible.

In July 2019, a Chinese study reported the development of a 19 SNP PRS for the prediction of lung cancer risk that had been prospectively validated in a cohort of more than 95 000 subjects.^123^ The study demonstrated that the PRS was better at lung cancer risk prediction than age and pack-year history alone; the 10% of the cohort with the highest genetic risk were 1.96 times more likely to develop lung cancer compared with the lowest risk 10%. It also showed that light smokers at high genetic risk have comparable lung cancer risk with heavy smokers with intermediate genetic risk. Light smokers with low genetic risk had a similar lung cancer risk to non-smokers. The study found striking genetic heterogeneity between several lung cancer histological subgroups. It should be noted that this study did not test the PRS in an actual screening programme cohort, nor did it compare its predictive abilities with a full RPM. The PRS developed is also specific to a Chinese population. Despite these limitations, this study is the best demonstration yet of the utility of a PRS in lung cancer risk prediction.

### Next steps and implementation

The successful, robust validation of a PRS for lung cancer, particularly one that was prospectively validated in such a large cohort, is an important milestone in the field. However, substantial research is required before such a tool will be ready for clinical use. As more samples and datasets become available from lung cancer screening trials and pilots around the world, opportunities will emerge to further validate and augment the list of SNPs thought to be associated with lung cancer risk. This will provide additional evidence to facilitate the construction of new PRS tools or to improve the tools presented in previous studies. The high-risk nature of individuals passing through screening programmes provides fertile ground for recruitment to nested case–control or case–cohort studies due to significant exposure to risk factors and different cancer outcomes. Machine learning approaches may also be useful in identifying SNPs associated with cancer risk, as well as more complex gene–gene interactions.^124^ It is important that this research be replicated in a wide variety of populations; many SNPs associated with lung cancer risk are specific to a particular ethnicity, necessitating the development of a PRS calibrated to the population it is intended to be used in. Developing PRS tools in diverse populations is important to ensure that the use of PRS in screening selection does not exacerbate health inequalities.^125^ Furthermore, many SNPs are associated with specific lung cancer histological subtypes. PRS construction must ensure that there is a sufficiently diverse array of SNPs on the panel to predict several types of lung cancer.

Following its construction, a PRS tool must be externally validated (ideally prospectively) in combination with the RPMs currently used in screening programmes to ensure that the PRS improves the predictive ability of the model. Some of the variables included in RPMs such as family history and tobacco smoke exposure might already be accounting for a portion of the risk impact conferred by genetic variants. Considering genetic risk factors in combination with demographic and lifestyle risk factors and testing them in an actual screening populations (such as has been done with the BOADICEA and Tyrer-Cuzick breast cancer RPMs^126 127^) ensures that personal risk is not overestimated and that the genetic component of the RPM has independent utility in a screening selection context.

Once a PRS tool integrated into an RPM has been shown to improve predictive ability, practical considerations relating to clinical implementation must be considered. Several biomarker studies embedded within lung cancer screening trials and programmes have provided evidence for the acceptability of blood collection from participants within screening settings and that a pipeline for blood storage and transportation, as well as the subsequent extraction of DNA and genotyping, is feasible.^128–130^ While good proof of concept, only establishing a participant’s PRS after their initial contact with the screening service means that it could not be used to inform screening eligibility at their initial assessment; the PRS could still be used to inform screening interval or to exclude low-risk individuals from further scans, but this limits the potential utility and effectiveness of the PRS. Asking participants to attend a separate clinic some time prior to screening for blood extraction would solve this issue but may reduce uptake and compliance among the target population. The genetic testing of saliva, rather than blood, could be an effective solution to this implementation challenge, as mailed collection kits can be returned by the participants for genotyping prior to any in-person contact with the screening service. Saliva collection has been shown to be acceptable to participants and a viable source of DNA for genotyping in several screening studies.^131–133^

Appropriate genetic counselling infrastructure must be implemented for PRS to become a routine tool for screening selection. An individual’s understanding of disease risk in general, how polygenic factors influence their risk and what impact this knowledge has on health behaviour and anxiety are all important psychological considerations that need addressing prior to clinical implementation. Research examining patient interest in PRS testing in other disease areas has revealed broadly positive attitudes^134 135^; patients also seem to receive their genetic risk score without significant distress or anxiety and are able to recall the information accurately.^136 137^ Development of tools for counselling patients in polygenic risk is ongoing.^138^ This research will need to be replicated in lung cancer screening populations, particularly considering that those at high risk of lung cancer often live in deprived areas, have low educational attainment and may have limited health literacy.^56^

Ultimately, the routine adoption of a PRS tool within a lung screening programme will depend on its clinical impact and cost-effectiveness. An effective PRS might reduce the total number of people eligible for screening or reduce the frequency of screening. It might also favour the selection of those who have a lower smoking exposure and therefore a lower burden of comorbidity who have ‘more to gain’ from screening.^139^ To reduce the cost of the test, the PRS could be targeted at those close to the risk threshold (above and below) rather than being used more broadly. Formal cost-effectiveness analyses would be required to determine the best approach within the setting of a lung cancer screening programme, as has been performed in other disease areas.^48 140 141^

## Conclusion

In an editorial following the publication of the NELSON trial results, Duffy and Field state: ‘*With the NELSON results, the efficacy of low-dose CT screening for lung cancer is confirmed. Our job is no longer to assess whether low-dose CT screening for lung cancer works: it does. Our job is to identify the target population in which it will be acceptable and cost-effective*’.^142^ Research in recent years has demonstrated that genetic factors, in particular the development and integration of polygenic risk scores into risk prediction models, could play a crucial role in augmenting the identification of the target population for lung cancer screening. There is an urgent need to construct a PRS that demonstrably improves risk prediction in an actual screening cohort over-and-above current RPMs, in a variety of populations and for several lung cancer histological subtypes. This could result in a lower rate of overdiagnosis and false-positive results in future screening programmes^39 143^ as well as their improved efficiency. Supplementary studies relating to the practical implementation of genetic testing in a lung cancer screening setting (including cost-effectiveness analysis and patient acceptability) will become important as the field develops further.
